# The genomic landscape of sensitivity to arsenic trioxide uncovered by genome-wide CRISPR-Cas9 screening

**DOI:** 10.3389/fonc.2023.1178686

**Published:** 2023-05-12

**Authors:** Jun-Zhu Chen, Li-Na Wang, Xue-Qun Luo, Yan-Lai Tang

**Affiliations:** Department of Pediatrics, The First Affiliated Hospital, Sun Yat-sen University, Guangzhou, Guangdong, China

**Keywords:** arsenic trioxide, CRISPR-Cas9 screening, KEAP1, leukemia, virtual screening

## Abstract

**Introduction:**

Arsenic trioxide (ATO) is a promising anticancer drug for hematological malignancy. Given the dramatic efficacy of acute promyelocytic leukemia (APL), ATO has been utilized in other types of cancers, including solid tumors. Unfortunately, the results were not comparable with the effects on APL, and the resistance mechanism has not been clarified yet. This study intends to identify relevant genes and pathways affecting ATO drug sensitivity through genome-wide CRISPR-Cas9 knockdown screening to provide a panoramic view for further study of ATO targets and improved clinical outcomes.

**Methods:**

A genome-wide CRISPR-Cas9 knockdown screening system was constructed for ATO screening. The screening results were processed with MAGeCK, and the results were subjected to pathway enrichment analysis using WebGestalt and KOBAS. We also performed protein-protein interaction (PPI) network analysis using String and Cytoscape, followed by expression profiling and survival curve analysis of critical genes. Virtual screening was used to recognize drugs that may interact with the hub gene.

**Results:**

We applied enrichment analysis and identified vital ATO-related pathways such as metabolism, chemokines and cytokines production and signaling, and immune system responses. In addition, we identified KEAP1 as the top gene relating to ATO resistance. We found that KEAP1 expression was higher in the pan-cancer, including ALL, than in normal tissue. Patients with acute myeloid leukemia (AML) with higher KEAP1 expression had worse overall survival (OS). A virtual screen showed that etoposide and eltrombopag could bind to KEAP1 and potentially interact with ATO.

**Discussion:**

ATO is a multi-target anticancer drug, and the key pathways regulating its sensitivity include oxidative stress, metabolism, chemokines and cytokines, and the immune system. KEAP1 is the most critical gene regulating ATO drug sensitivity, which is related to AML prognosis and may bind to some clinical drugs leading to an interaction with ATO. These integrated results provided new insights into the pharmacological mechanism of ATO and potentiate for further applications in cancer treatments.

## 1 Introduction

Arsenic trioxide (ATO) is an established agent in treating acute promyelocytic leukemia (APL), a hematological malignancy with the unexpected fusion promyelocytic leukemia-retinoic acid receptor α (PML-RARa) as molecular characterization (1). ATO degrades the existing PML-RARa and triggers cell death by apoptosis (2) and autophagy (3–6). Another target, Fms-like tyrosine kinase 3 gene internal tandem duplication (FLT3-ITD), has been confirmed to be downregulated by ATO, resulting in autophagic degradation and cell cytotoxicity (7–9). Apart from targeting these abnormal fusions or mutated proteins, ATO has been proven with a pro-oxidant effect (10) in treating cancer cells and can rescue structural p53 mutations (11), providing a promising anticancer therapy in other hematological diseased and solid tumors. However, the current trials revealed that ATO was less effective in other types of cancers compared with its above 90% cure rate in APL (12–22). This unsatisfactory result may be related to the insufficient concentration in solid tumor sites and the primary or acquired resistance to ATO. The solutions for the former problem mainly focus on improving the drug delivery system by nanoparticles from exogenous materials (23, 24) or biomimetic nanocarriers such as ferritin (25). As for the resistance caused by genetic events, most studies have recognized PML mutations as a primary resistant mechanism (1, 26), and treatments targeting PML-RARa fusion protein showed a synergistic antileukemic effect in combination with ATO (27, 28). The epigenetic changes induced by ATO also made them valuable targets for overcoming resistance (29). Unfortunately, these results cannot explain the resistance in other cancers which do not harbor such mutations. Furthermore, the current combination methods of ATO concentrate more on regulating the reactive oxygen species (ROS) contents to overcome resistance with limited effect (26, 30–33). Therefore, a more systematic landscape to describe genetic events and identify potential targets or entrances of ATO is required to gain a fully comprehensive understanding of the pharmacology of ATO and further overcome resistance.

Genome-wide CRISPR-Cas9 screening is a revolutionary method appearing in biomedicine in the last decade. Based on the CRISPR-Cas9 system, this screening system effectively creates a genome-wide knockout cell library, and the cells are further challenged with certain drugs, toxins, or virals. The depleted genes will contribute to a specific phenotype, such as cell death or survival, and thus are selected as essential targets of chemical and biological agents. Taking advantage of its high throughput (nearly 20,000 genes at one time) and unbiased nature, the CRISPR-Cas9 screening has emerged as a handy technique for identifying the genes or pathways relevant to the sensitivity or resistance of existing treatments. The determinants of cell response to a list of anticancer drugs have been identified successfully through this method, including traditional chemo agents [Ara-C (34), etoposide (35), lenalidomide (36), and cisplatin (37), e.g.] or small molecular-targeted drugs such as vemurafenib (38–40), panobinostat (41), alisertib (42), ceralasertib (43), olaparib (44–46), selumetinib (47), and trametinib (47).

The Kelch-like ECH-associated protein 1 (KEAP1) is the central cellular defense module against oxidative stresses. As a cysteine thiol-rich sensor of redox stress, KEAP1 binds to and represses NF-E2-related factor 2 (NRF2) through the ubiquitin-26S proteasome pathway in quiescent but releases it on exposure to stress. NRF2 then induces the transcription of several cytoprotective genes to defend against redox insults such as ROS (48). Notably, the production of ROS contributes to the cytotoxicity of cancer cells, indicating the significant role KEAP1 plays in therapeutic resistance. The loss-of-function mutations in KEAP1 confer radiation resistance in non-small cell lung cancer (NSCLC) (49, 50). Several studies have confirmed that the KEAP1–NRF2 complex renders chemoresistance in treatments for colorectal cancer, esophageal squamous cancer, and pancreatic cancer (51–53). The underlying mechanism of resistance relates to the robust production of detoxification molecules, including NAD(P)H quinone dehydrogenase 1 (NQO1), glutathione-S-transferase (GST), and glutathione (GSH), which conjugate chemo agents like cisplatin and then excreted (54, 55). Therefore, KEAP1 is a crucial regulator in cell response to cancer therapy.

In this study, we performed a genome-wide CRISPR-Cas9 loss-of-function screening on ATO to identify the essential genes and pathways in cell response to ATO. The enriched screening results showed that the genes related to metabolism, chemokines and cytokines production and signaling, and immune system responses might contribute to cell death induced by ATO in addition to the effect of ROS. These findings provided a thorough understanding of mechanistic effects and a broader view of the potential application of ATO.

## 2 Materials and methods

### 2.1 Cells and plasmids

The 293T and HAP1 cells used in screening were kept in our laboratory. Dulbecco’s Modified Eagle Medium (DMEM, HyClone) was used for 293T maintenance, and Iscove’s Modified Dulbecco’s Medium (IMDM, HyClone) for HAP1 cells. The mediums were supplemented with 10% fetal bovine serum (FBS, NEWZERUM) and 1% penicillin/streptomycin (HyClone). All cells were maintained in a humidified, 5% CO2 incubator at 37°C. The plasmids psPAX2 (RRID: Addgene_12260) and pMD2.G (RRID: Addgene_12259) were obtained via Addgene. Human Brunello CRISPR knockout pooled library was a gift generously provided by David Root and John Doench (RRID: Addgene_73179) (40).

### 2.2 Genome-wide CRISPR-Cas9 screening

We followed the protocol published by Zhang Feng et al. (56) to conduct the experiments. We utilized the sgRNA library from David Root and John Doench (40), the Brunello library. The plasmid library contains 76,441 gRNAs targeting 19,114 human genes. We used 293T cells, helper plasmids (psPAX2 and pMD2.G), and the plasmid library to generate a lentivirus pool. The culture media of HAP1 cells were replaced with fresh media containing 8 μg/ml polybrene (Beyotime) before lentivirus transfection. To maintain the representativity of the library, we transfected the HAP1 cells at a representation of 500 cells per sgRNA. The multiplicity of infection (MOI) was 0.3 in this study to ensure that each cell obtained one sgRNA. Cells were then cultured under 1 μg/ml puromycin (Beyotime) for 7 days and split into two groups with ATO (2 μM, SLPHARM) or vehicle (DMSO) treatment for 14 days. After propagating to identify sgRNAs enriched or depleted, the genomic DNAs of harvested cells were extracted by TIANamp Genomic DNA Kit (#DP304, TIANGEN) and PCR amplified. The PCR fragments were gel-purified utilizing E.Z.N.A.^®^Gel Extraction Kit (#D2500, OMEGA) and sequenced by Novogene (Beijing, China). The genes enriched or depleted were identified by the model-based analysis of genome-wide CRISPR-Cas9 knockout (MAGeCK) software (https://sourceforge.net/projects/mageck/) with default parameters. The MAGeCK-RRA algorithm counted each gene’s Robust Rank Aggregation (RRA) score. Essential genes were identified at *p* < 0.05 and |log_2_ fold change| ≥1 by comparing ATO-treated sgRNA with vehicle-treated sgRNA. Sequencing data are deposited at Gene Expression Omnibus (GSE218982).

### 2.3 Functional enrichment analysis

WEB-based GEne SeT AnaLysis Toolkit (WebGestalt, v.2019, RRID: SCR_006786) is a widely used functional enrichment analysis web tool (57–60). In this study, WebGestalt was used to conduct Gene Ontology (GO) enrichment analysis (daily build accessed on 01/14/2019), including biological process (BP), cellular component (CC), and molecular function (MF) categories. KEGG Orthology Based Annotation System (KOBAS, v.3.0, RRID: SCR_006350) is a web server for gene functional annotation and enrichment (61). The enrichment analysis is based on the ORA (overrepresentation analysis) method, a simple and frequently used gene set enrichment method using the Hypergeometric test and Fisher’s exact test. False discovery rate (FDR) correction (*Q*-value) was performed by Benjamini and Hochberg method. In this study, we used KOBAS and chose three databases, namely, REACTOME (62), Protein ANalysis THrough Evolutionary Relationships (PANTHER) (63, 64), and Kyoto Encyclopedia of Genes and Genomes (KEGG) (65), for cell informative pathway enrichments. The cutoff value was indicated in figure legends.

### 2.4 Construction of the protein–protein network

The Search Tool for the Retrieval of Interacting Genes/Proteins (STRING, v.11.5, RRID: SCR_005223) database (66) and Cytoscape (v.3.9.1, RRID: SCR_003032) software (67) were used to establish and visualize a protein–protein interaction (PPI) network of depleted and enriched genes identified by the genome-wide CRISPR-Cas9 screening of ATO. The plugin Molecular COmplex DEtection (MCODE) (68) in Cytoscape was applied to calculate the degree of each protein and recognized the key modules and hub genes with default parameters (degree cutoff at 2, node score cutoff at 0.2, K-core at 2, and max depth at 100). The hub genes of the top cluster identified by MCODE were then analyzed by the plugin Cluego (69) and CluePedia (70) for REACTOME pathway enrichment (25.05.2022 version).

### 2.5 Gene expression profiles, correlation analysis, and survival analysis

TNMplot (https://tnmplot.com/analysis) is a web analysis tool focused on differential gene expression in tumor, normal, and metastatic tissues (71). This database utilizes RNA-seq and clinical data from GTex, TCGA, and TARGET databases to analyze gene expression comparison in a selected tumor or pan-cancer. This study applied this tool to investigate the KEAP1 gene expression in acute lymphoblastic leukemia (ALL) compared with normal tissues utilizing TARGET (https://ocg.cancer.gov/programs/target), a database with comprehensive molecular characterization and clinical prognosis of childhood cancers. We also used TNMplot to generate the KEAP1 gene expression profiles in pan-cancer.

GEPIA2 (RRID: SCR_018294) is a newly developed interactive web server for analyzing the RNA-seq data of samples from the GTex and TCGA projects and is widely used for patient survival analysis (72). This research used this web tool to identify whether the KEAP1 gene expression was related to prognosis in acute myeloid leukemia (AML) patients.

The half maximal inhibitory concentration (IC50) values of ATO in different cell lines were collected from our former study (8) and Cellminer (NCI-60 project) (73). The KEAP1 expression per kilobase per million reads log_2_(FPKM + 1) values were collected from the NCI-60 project (74) and Depmap expression public 22Q4 (https://depmap.org/portal/). The correlation analyses were performed using Pearson correlation.

### 2.6 Virtual screening

A total of 2,506 FDA-approved drugs from DRUGBANK (RRID: SCR_002700) were involved in the virtual screening. The ligand database was energy minimized using MMFF force field (steepest descent) in the open-source OpenBabel software package (http://openbabel.org/) and saved in mol2 format. The compound library is then pre-processed and saved in pdbqt format using prepare_ligand4.py from AutoDock tools (https://ccsb.scripps.edu/mgltools/).

The crystal structure of the human KEAP1 BTB domain and Kelch domain was obtained from RCSB PDB (RRID: SCR_012820, https://www.rcsb.org, PDB ID: 7EXI, 6TYM) (75). Proteins were pre-processed in PyMol (http://pymol.org/2/, RRID: SCR_000305) (76) to remove waters and ligands and in AutoDock tools to add polar hydrogens, charges by Kollman charge, and saved in pdbqt format. The grid box of the KEAP1 kelch domain was defined by ligand in 6TYM. The binding pocket of the KEAP1 BTB domain was predicted by DeepSite (https://www.playmolecule.com/deepsite/). The grid box was defined by employing AutoDock tools’ Grid setting feature.

We used Smina for molecular docking, an open-source molecular docking software based on AutoDock Vina (https://vina.scripps.edu) (77–79). Molecular docking parameters in this study are as follows: exhaustiveness = 8, num_modes = 10, energy_range = 3, min_rmsd_filter = 1. After the complete execution of docking, the top 20 minimized affinity ligands were obtained. Subsequently, the neural-network-based scoring function (NNScore2) of those 20 ligands was calculated, and the top 10 compounds for each domain with predictive IC50 value were selected as potential candidates targeting KEAP1 (80). We used PyMol to visualize the docking position.

### 2.7 Data visualization and statistical analysis

**Figure 1:**
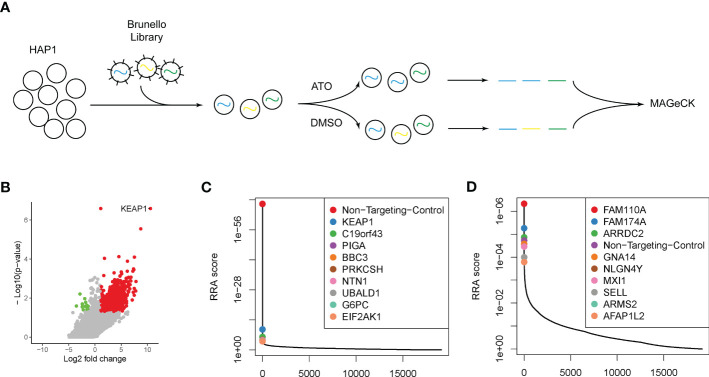
Genome-wide CRISPR-Cas9 screening identified key modulators attributing to ATO sensitivity. **(A)** The schematic outline of the genome-wide CRISPR-Cas9 screening. **(B)** The volcano plot of genes significantly enriched (red dots) or depleted (green dots) in ATO treatment groups compared with vehicle. The cutoff threshold was |log_2_ fold change| ≥1 and *P* < 0.05. **(C)** The positively selected genes in ATO treatment groups compared with vehicle. **(D)** The negatively selected genes in ATO treatment groups compared with vehicle.

**Figure 2:**
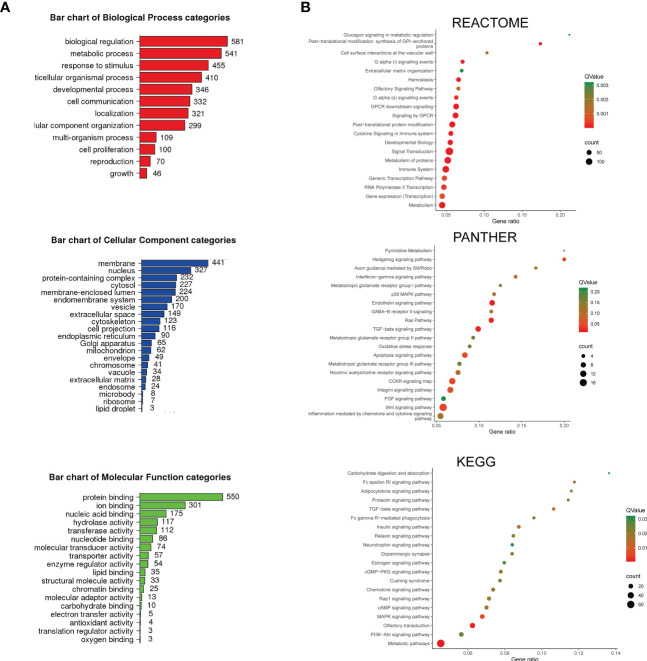
The enrichment analysis of depleted and enriched genes was identified by screening. **(A)** The bar charts of Gene Ontology (GO) enriched terms. The enrichment analysis was performed and plotted by WebGestalt. The top most relevant GO terms with an adjusted *P* < 0.05 were shown in the charts. **(B)** Pathway enrichment analysis. The enrichment analysis was performed by KOBAS. REACTOME, PANTHER, and KEGG-enriched terms were presented separately. The color demonstrated the *Q*-value of each pathway, and the size of the dots demonstrated the count of genes related to the pathway.

**Figures 1B**, **2B**, and **4A** were plotted by ImageGP (http://www.ehbio.com/ImageGP/index.php/Home) (81). The other figures were visualized as indicated in figure captions. The methods for the significance test were described in related texts or figure legends.

## 3 Results

### 3.1 Genome-wide CRISPR-Cas9 screening of arsenic trioxide in HAP1 cells

To recognize the essential genes and pathways related to the sensitivity or resistance of ATO, we performed a genome-wide CRISPR-Cas9 knockout loss-of-function screen in the HAP1 cell line (**Figure 1A**). We chose this cell model according to its haploid nature, so that the integrity sister chromatid would not mask the knockout effect. The Brunello sgRNA library has been previously described and targets 19,114 human genes with 76,441 distinct sgRNAs (40). The transduced cell library was then exposed to 2 μM ATO (IC50 of HAP1 cells to ATO) or DMSO as a control group. The genomic DNAs were collected, PCR amplified, and sequenced. Using the MAGeCK tool (82), we examined the data to determine the relative enrichment or depletion of each sgRNA compared with the control groups. We mapped the genes whose deletion resulted in ATO sensitivity (results shown in **Supplementary Table S1**). Nine hundred eighty-four genes were recognized as contributors to the sensitivity to ATO with |log_2_ fold change| ≥1 and *p* < 0.05 (**Figure 1B**). Among these genes, we identified 970 positively selected genes whose deletion contributed to the resistance to ATO or were potential targets as the entrance of cell responses to ATO (**Figure 1B**, red dots). The top genes ranked by RRA score were listed and plotted (**Figure 1C**). These genes included Kelch ECH associating protein 1 (KEAP1), a sensor of oxidative stress, which has been confirmed to contribute to the progression and resistance to chemo- and radiotherapy in many types of cancers (83, 84). BBC3, coding BCL2-binding component involved in p53 signaling apoptosis, was also highly selected. Genes involved in the endoplasmic reticulum (ER) stress were also found in the top-ranked candidates, PRKCSH and EIF2AK1. Other genes positively selected in this study, including C19orf43, PIGA, NTN1, UBALD1, and G6PC, have not yet been related to cell response to ATO. Meanwhile, we recognized 15 negatively selected genes under the treatment of ATO (**Figure 1B**, green dots). The deletion of this group of genes can improve the sensitivity to ATO and are potential targets in combination with ATO to overcome the resistance to ATO. The top 10 selected genes ranked by RRA score were listed in **Figure 1D**. The genes involved in cell cycle regulation should be identified in negativity selection, because the deletions of this group of genes were bound to induced cytotoxicity. In this study, we recognized FAM110A, localized to centrosomes and accumulated at the microtubule organization center, and AFAP1L2, regulating the mitotic cell cycle, as cell cycle–related genes in response to ATO. We also identified several genes functioning in cell adhesion. GNA14, encoding a member of the guanine nucleotide binding, or G protein family, was a well-known target in hepatocellular carcinoma and vascular tumors (85, 86). The protein encoded by NLGN4Y (Neuroligin 4 Y-Linked) is presented at the postsynaptic side of the synapse (87). Another vital cell surface adhesion molecule, SELL (Selectin L, or LAM-1), mediated cell adhesion by binding to glycoproteins on neighboring cells in a calcium-dependent manner, a key regulator in viral infection (88–91). In addition, a tumor suppressor negatively regulating MYC function in lung cancer, MXI1, was also identified in the selection (92). The exact function of the other genes in the top rank, FAM174A, ARRDC2, and ARMS2, remained unknown in ATO or cancer research.

### 3.2 Enrichment analysis of pathways relating to arsenic trioxide sensitivity

To understand the overview mechanism of cell response in sensitivity to ATO, we performed enrichment analysis on the 984 genes recognized with |log_2_ fold change| ≥1 and *P* < 0.05 in screening. First, we applied WebGestalt for GO analysis to annotate genes products and characteristics (**Figure 2A**). GO terms with a *P* < 0.05 were displayed. The top GO enrichment items were classified into three functional categories, namely, BP (12 items, **Figure 2A**, red panel), CC (21 items, **Figure 2A**, blue panel), and MF (18 items, **Figure 2A**, green panel). These genes were mainly enriched in biological regulation, metabolic process, response to stimulus, multicellular organismal process, and developmental process referring to BP. The most enriched CC annotations included membrane, nucleus, protein-containing complex, cytosol, and membrane-enclosed lumen. As for MF, these genes were significantly involved in protein binding, ion binding, nucleic acid binding, hydrolase activity, and transferase activity.

Next, we utilized KOBAS and performed the pathway enrichment analysis. We chose three databases (REACTOME, PANTHER, and KEGG) for their enriched tactics and annotations (**Figure 2B**). REACTOME focused on the reactions and grouped entities into a network based on their reaction participation. Most of the enriched pathways in this study were linked with metabolism, including glucagon signaling in metabolic regulation and metabolism of proteins, followed by GPCR downstream signaling events, cytokine signaling, immune system, and transcription events. PANTHER clustered proteins through evolutionary and functional relationships. The enrichment study using this database revealed that the cell response to ATO was significantly relevant to pyrimidine metabolism, Hedgehog signaling pathway, and interferon−gamma signaling pathway. In addition, the pathways involving the metabotropic glutamate receptor groups and a broad spectrum of chemokine and cytokine signaling (p38 MAPK, Ras, TGF−beta, FGF, and Wnt) were also enriched. It should be noted that the oxidative stress response and apoptosis signaling pathway were also included, which were consistent with the top-rank gene result in this study and the known mechanism. KEGG signaling pathway annotations, one of the most frequently used open databases integrating genomic and systemic functional information, were also analyzed. The enriched pathways were associated with a series of metabolism processes, including carbohydrate digestion and absorption, adipocytokine, insulin, estrogen signaling pathways, and Cushing syndrome. The immune-related pathways (Fc epsilon RI signaling, Fc gamma R−mediated phagocytosis, and neurotrophin signaling pathway) and chemokine and cytokine signals (TGF−beta, cGMP−PKG, Rap1, MAPK, and PI3K-Akt) were also included. Therefore, these results demonstrated that the sensitivity of ATO could be affected by varied cell responses relating to metabolism, chemokines and cytokines, and the immune system, indicating that ATO was an anticancer candidate with broad targets and functions.

### 3.3 Hub genes recognized by a protein–protein interaction network

**Figure 3:**
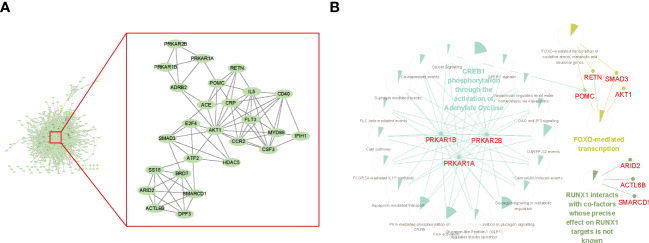
Protein–protein interaction (PPI) network among depleted or enriched genes. **(A)** The hub genes of the PPI. The PPI network was generated by the STRING database with default settings and was built by Cytoscape. The cluster of hub genes within the red frame was calculated using MCODE in Cytoscape with the default setting. **(B)** The top enriched REACTOME pathways of the hub genes. The enrichment analysis was performed and visualized by Cytoscape using the plugin Cluego and CluePedia. The sector graphs indicated the portion of related genes compared with all the enriched pathway genes.

To understand the relationship between genes recognized in the screening, we performed a PPI network analysis. PPI assessed the physical and functional connections between the proteins of target genes to provide new aspects of the selected genes. The PPI network was illustrated using the STRING website and visualized in Cytoscape software (**Figure 3A**, left). The stability of the entire network mostly depends on nodes (genes in this context) with a higher degree of connectivity, that is, the core module. To recognize the significant gene modules in this network, we used the plugin MCODE with default parameters and identified a cluster with the highest hub score of 6.24 (**Figure 3A**, right). This essential module contained 26 hub genes, including important transcription factors (ATF2 and E2F4) and kinases (FLT3 and AKT1). To explore the functional relationships between hub genes, we performed REACTOME pathway enrichment analysis with the plugins Cluego and CluePedia (**Figure 3B**). The hub genes were mainly enriched in CREB1 phosphorylation by activating Adenylate Cyclase, FOXO-mediated transcription, and RUNX1 interactions. These results revealed that ATO was significantly related to transcription factor activity.

### 3.4 KEAP1 as a critical modulator in sensitivity to arsenic trioxide

**Figure 4:**
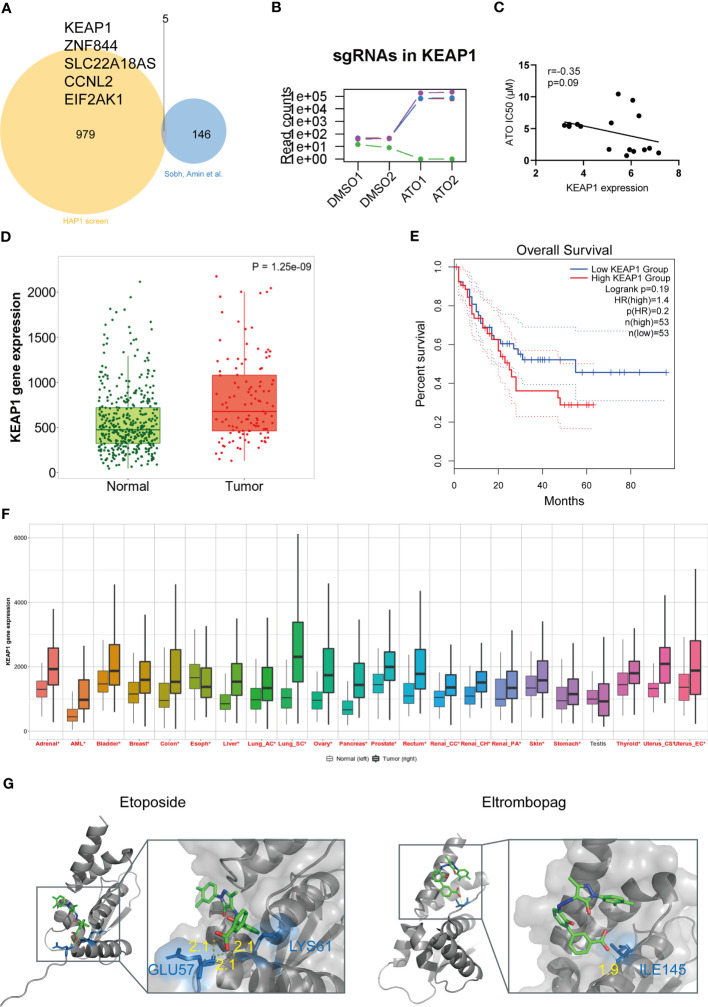
KEAP1 was a vital target of ATO with clinical significance in pan-cancer. **(A)** The Venn diagram of overlapping genes between two studies plotted by ImageGP. **(B)** The read counts of sgRNAs targeting KEAP1 in screening. **(C)** The correlation analysis of KEAP1 gene expression and sensitivity to ATO. Gene expression showed the log_2_ (FPKM + 1) values. The correlation analyses were performed using Pearson correlation. **(D)** The box plot derived from KEAP1 expressions data for acute leukemias of ambiguous lineage (ALAL) compared with normal samples from non-cancerous pediatric tissues. The plot was generated by TNMplot. Significant differences were tested by the Mann-Whitney *U* test. **(E)** The Kaplan–Meier survival plot comparing overall survival (OS) with high and low KEAP1 expression in AML patients generated using GEPIA2. Significance was tested by log-rank test. **(F)** The pan-cancer gene expression profiles of KEAP1 generated by TNMplot. Significant differences (*P* < 0.05) by the Mann-Whitney *U* test were marked with red*. **(G)** The docking poses of two drugs. The yellow dot lines indicate hydrogen bonds. The blue sticks represent residues.

A similar genome-wide CRISPR-Cas9 screening focused on ATO has been performed by Amin Sobh et al. They utilized a different sgRNA library (GeCKO v2 sgRNA library containing 65,383 sgRNA targeting approximately 19,000 human genes) and cell lines [K562, a Chronic Myelogenous Leukemia (CML) cell line derived from human bone marrow] and identified a total of 151 genes with significance (93). We speculated that the essential genes relating to ATO sensitivity should be recognized in these two screens and compared the enriched or depleted genes in our study with the another. The Venn diagram showed that the overlapping genes were KEAP1, ZNF844, SLC22A18AS, CCNL2, and EIF2AK1 (**Figure 4A**). Among these five genes, KEAP1 was top ranked in both two studies. It seemed that the deletion of KEAP1 significantly damaged the cell death to ATO and may be a key entrance target of ATO to trigger cell responses. We reviewed the counts of sgRNAs targeting KEAP1 in different groups and found that three of four sgRNAs were significantly enriched in ATO-treated groups (**Figure 4B**). In addition, the IC50 values of ATO were negatively correlated with KEAP1 expression in leukemic cell lines (**Figure 4C**). These results illustrated that KEAP1 did play a role in cell sensitivity to ATO.

We then investigated the clinical prognosis effect of KEAP1. Given that ATO was primarily used in hematological malignance instead of another disease, we first assessed the KEAP1 expressions in leukemia patients from the TARGET database, which contained genetic characteristics and clinical information of pediatric acute ALL patients. We found that the KEAP1 expression was higher in acute leukemias of ambiguous lineage (ALAL) patients compared with normal (*p* < 0.05) (**Figure 4D**). However, the expression did not affect the overall survival (OS) or disease-free survival (DFS) (data not shown). In addition, no expression difference was shown in other types of leukemia from TARGET (data not shown). Then, we tested the effect on AML from the TCGA database utilizing GEPIA2. The high expression of KEAP1 predicted poor OS in AML patients (**Figure 4E**). Finally, we examined the expression in pan-cancer from TCGA and found that all the tumors except for the testis were of higher KEAP1 expression compared with normal tissues (**Figure 4F**). These results suggested that ATO may be a potential therapy in treating pan-cancer through a mechanism involving KEAP1.

Considering that anticancer therapy was usually performed in combination, we were interested in what drugs may interact with KEAP1 and be a potential sensitizer or inhibitor when combined with ATO. To uncover these drug-gene interactions, we performed virtual screening for KEAP1 and screened for 2,506 FDA-approved drugs from DRUGBANK. The drugs with the highest affinity to the domain of KEAP1 were listed (**Table 1**). Among these drugs, we were particularly interested in etoposide and eltrombopag. Etoposide (VP-16) is a topoisomerase II inhibitor and an essential chemotherapeutic agent. It is the frontline drug used against several types of cancer, including pediatric ALL (94) and AML (95). Moreover, eltrombopag is a thrombopoietin (TPO) receptor agonist for the treatment of aplastic anemia (96) and chemotherapy-induced thrombocytopenic patients (97, 98). Therefore, these drugs may interfere with the therapeutic effect of ATO during cancer treatment. The docking poses of these two drugs with the KEAP1 BTB domain were shown in **Figure 4G**. These results indicated a potential interaction with ATO through KEAP1.

**Table 1:** The results of the virtual screening of KEAP1.

Domain	Drugbank ID	Name	CAS_number	Minimized Affinity	NNS2_SCORE
Kelch domain	DB00278	Argatroban	74863-84-6	-11.0561628	12.05 nM
	DB00966	Telmisartan	144701-48-4	-10.573246	2.49 nM
	DB01126	Dutasteride	164656-23-9	-10.6675119	1.84 nM
	DB09074	Olaparib	763113-22-0	-11.1431427	7.96 nM
	DB09280	Lumacaftor	936727-05-8	-10.7092714	10.16 nM
	DB09372	Tannic acid	1401-55-4	-10.780798	17.21 nM
	DB11262	Bisoctrizole	103597-45-1	-10.5385208	4.29 nM
	DB11986	Entrectinib	1108743-60-7	-10.8520021	488.86 pM
	DB14703	Dexamethasone metasulfobenzoate	16978-57-7	-11.2371359	2.74 nM
	DB15982	Berotralstat	1809010-50-1	-11.0051241	25.75 nM
BTB domain	DB00444	Teniposide	29767-20-2	-7.10783052	7.76 µM
	DB00759	Tetracycline	60-54-8	-6.6632781	23.28 µM
	DB00773	Etoposide	33419-42-0	-6.87911749	4.51 µM
	DB00820	Tadalafil	171596-29-5	-6.69839001	8.29 µM
	DB06210	Eltrombopag	496775-61-2	-6.55944538	913.0 nM
	DB08995	Diosmin	520-27-4	-6.6483264	17.11 µM
	DB09280	Lumacaftor	936727-05-8	-6.80042887	2.33 µM
	DB12001	Abemaciclib	1231929-97-7	-6.57445669	829.46 nM
	DB14703	Dexamethasone metasulfobenzoate	16978-57-7	-6.78505754	516.76 nM
	DB15690	Fluoroestradiol F-18	94153-53-4	-6.6101265	8.64 µM

## 4 Discussion

Our work uncovered a vast landscape on which genetic perturbations attributed to the sensitivity to ATO. ATO attenuates the induction of ROS (99). The ability of ATO to promote ROS formation and ER-mitochondria cross-response has been confirmed by a series of experimental evidence in normal cells or cancer cells (100, 101), eventually leading to apoptosis or autophagy. KEAP1, the top-ranked gene in this study, was a key regulator in response to oxidative stress. It interacts with NRF2 and dissociates after sensing redox, leading to the transportation of NRF2 from the cytoplasm to the nucleus. This reaction results in a cascade of events against the redox stress. Our result suggested that KEAP1 is the most vital regulator in response to ATO instead of other genes involved in oxidative reactions. The loss-of-function of KEAP1 causes robust transportation of NRF2 and activates the antioxidant reactions, defending cells against ATO. Previous studies have also clarified that the NRF2-associated activation and downstream GSH biosynthesis accelerated arsenic efflux and ameliorated the cytotoxicity of ATO (83). The exact underlying mechanism remained to be explored. As indicated in this study, KEAP1 was differently expressed in tumors and related to the prognosis of certain types of cancers. It seems that ATO can be potentially applied to other cancers as a candidate therapy.

The other enriched pathways in this study were consistent with the current understanding of sensitivity and resistance to ATO. The metabolic process was enriched in GO and pathway analysis. ATO has been identified with an inhibitory effect on the glycolytic pathway (102). Lower glucose uptake and the distinct metabolical pattern were recognized in ATO-resistant cell lines (33, 103). Abu Bakar Noraini et al. found that ATO altered lipids metabolites, particularly arachidonic acid and docosahexaenoic acid (104). We also linked chemokines and cytokines productions with ATO. The chemokine-induced differentiation syndrome is one of the most common causes of death in APL patients, formerly known as a retinoic acid syndrome (105). Nevertheless, further evidence showed that ATO could induce chemokine production as a single agent (106). Considering that ATO-induced apoptosis is mediated by PI3K/Akt signaling pathway (107–109), which was also identified in our study, the involvement of ATO in regulating chemokine and cytokine may be of wide spectrum and related to chemoattraction and inflammation. Another vital pathway enriched in this study was the immune system. We performed enrichment analysis in three different databases. REACTOME and PANTHER enriched the immune system pathway, and we identified interferon-gamma and CCKR as immune-related signaling pathways. Fc epsilon RI signaling and Fc gamma R−mediated phagocytosis were enriched by KEGG as well. The cross talk between ATO and the immune system has not been clarified yet. Srivastava, Ritesh K et al. reported that ATO regulated macrophage innate immune function via unfolded protein response (UPR) signaling and activating transcription factor 4 (ATF4) (110, 111). ATO also exerts its efficacy on regulatory T cells (112–114). Chen Jinfeng et al. recently demonstrated that ATO was highly immunogenic and increased antigenicity and adjuvanticity after preconditioning *ex vivo* (115). These corroborated observations shed light on the correlation between ATO and cancer immunity and a promising therapeutic agent for autoimmune diseases (116).

The haploid cells that we used as cell models in this study, HAP1 cells, suit the aim of genetics research. The HAP1 cell line contains only one copy of the genome and shows the unmasked phenotype of different variants. In addition, it has a rapid doubling time and is sensitive to transfection, making it a handy and revolutionary model for gene editing. It has been applied in screens on resistance to a spectrum of anticancer drugs (117–120). The screens in HAP1 cells draw drug-specific conclusions instead of a cell type–specific character, making the interpretation easier in pan-caner. As mentioned, Amin Sobh et al. screened ATO using a different sgRNA library and CML cell lines (93). Although both studies identified that the disruption of KEAP1 markedly increased ATO tolerance, the genes involved in selenocysteine metabolism were recognized and confirmed to be resistant in their study but not ours. These differences may partly be due to the different cell lines and thresholds that we employed.

Our research showed that CRISPR-based functional genomics screening could be utilized to understand the molecular processes influencing sensitivity to ATO. The results may have implications for using ATO in chemotherapy in situations other than treating APL. However, further studies are needed to validate the relevance of this study, especially in cell-specific contexts.

## Data availability statement

The datasets presented in this study can be found in online repositories. The names of the repository/repositories and accession number(s) can be found below: https://www.ncbi.nlm.nih.gov/geo/,GSE218982.

## Author contributions

J-ZC and Y-LT designed the study. J-ZC performed the experiments. L-NW and X-QL contributed to the study design and scientific discussions. J-ZC drafted the manuscript. X-QL and Y-LT revised the manuscript critically. All authors contributed to the article and approved the submitted version.
